# Supplementary material for: End Sequence Analysis Toolkit (ESAT) expands the extractable information from single-cell RNA-seq data
Source: Genome Res. 2016 Oct;26(10):1397–410. doi: 10.1101/gr.207902.116 (PMC5052061; doi:10.1101/gr.207902.116)
Supplement: Supplemental Material [file supp_gr.207902.116_Supp_Materials.tar.gz › ESAT-esat_v0.1/doc/html/classbroad_1_1core_1_1math_1_1_scan_statistics.html]

ESAT: broad.core.math.ScanStatistics Class Reference


ESAT


- Main Page
- Related Pages
- Packages
- Classes
- Files

- Class List
- Class Index
- Class Hierarchy
- Class Members

All Classes Namespaces Files Functions Variables Enumerator Pages

- broad
- core
- math
- ScanStatistics

Static Public Member Functions |
List of all members

broad.core.math.ScanStatistics Class Reference

|  |  |
| --- | --- |
| Static Public Member Functions | |
| static double | calculatePVal (int k, double lambda, double w, double T) |
|  | |
| static double | calculateApproximatePVal (int k, double lambda, double w, double T, double alpha) |
|  | |
| static double | poisson (int k, double lambda) |
|  | |
| static double | Fp (int k, double lambdaW) |
|  | |
| static double | FpWithBreaking (int k, double lambdaW, double a, double stopPoint) |
|  | |

## Member Function Documentation

|  |  |  |  |
| --- | --- | --- | --- |
| static double broad.core.math.ScanStatistics.calculateApproximatePVal | ( | int | *k*, |
|  |  | double | *lambda*, |
|  |  | double | *w*, |
|  |  | double | *T*, |
|  |  | double | *alpha* |
|  | ) |  |  |

static

Here is the call graph for this function:

|  |  |  |  |
| --- | --- | --- | --- |
| static double broad.core.math.ScanStatistics.calculatePVal | ( | int | *k*, |
|  |  | double | *lambda*, |
|  |  | double | *w*, |
|  |  | double | *T* |
|  | ) |  |  |

static

Params written by Jesse Aug 20, 2012 ... are these definitions correct?

Parameters
:   |  |  |
    | --- | --- |
    | k | Observed count |
    | lambda | # reads on chromosome / # non-masked bases on chromosome |
    | w | window size |
    | T | # non-masked bases on chromosome |

Returns

Here is the call graph for this function:

Here is the caller graph for this function:

|  |  |  |  |
| --- | --- | --- | --- |
| static double broad.core.math.ScanStatistics.Fp | ( | int | *k*, |
|  |  | double | *lambdaW* |
|  | ) |  |  |

static

Here is the call graph for this function:

Here is the caller graph for this function:

|  |  |  |  |
| --- | --- | --- | --- |
| static double broad.core.math.ScanStatistics.FpWithBreaking | ( | int | *k*, |
|  |  | double | *lambdaW*, |
|  |  | double | *a*, |
|  |  | double | *stopPoint* |
|  | ) |  |  |

static

Here is the call graph for this function:

Here is the caller graph for this function:

|  |  |  |  |
| --- | --- | --- | --- |
| static double broad.core.math.ScanStatistics.poisson | ( | int | *k*, |
|  |  | double | *lambda* |
|  | ) |  |  |

static

Here is the caller graph for this function:

---

The documentation for this class was generated from the following file:

- java/broad/core/math/ScanStatistics.java


---

Generated on Fri Aug 15 2014 15:10:53 for ESAT by  

 1.8.7
